# Supplementary figures and images for: Genome-Wide Scans and Transcriptomic Analyses Characterize Selective Changes as a Result of Chlorantraniliprole Resistance in Plutella xylostella
Source: Int J Mol Sci. 2022 Oct 13;23(20):12245. doi: 10.3390/ijms232012245 (PMC9603363; doi:10.3390/ijms232012245)

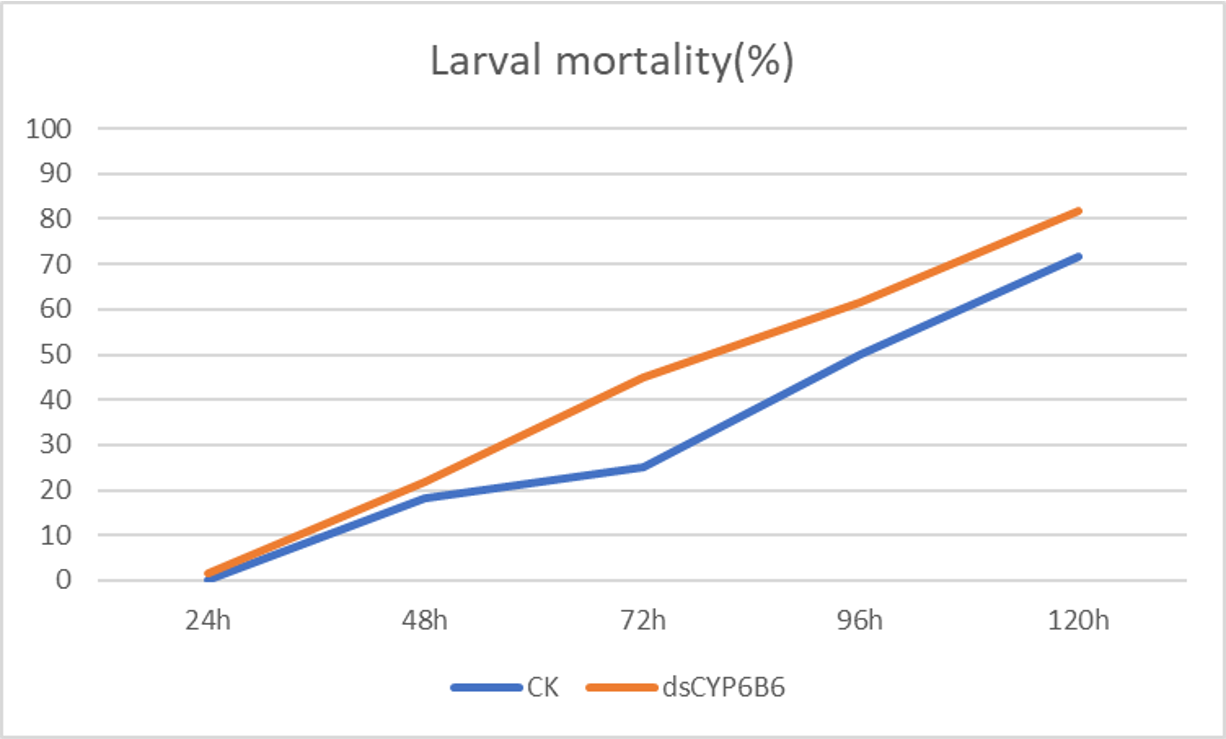

Supplement: Supplementary file 1 [file ijms-23-12245-s001.zip › Supplementary Material/S10_Fig.tif]

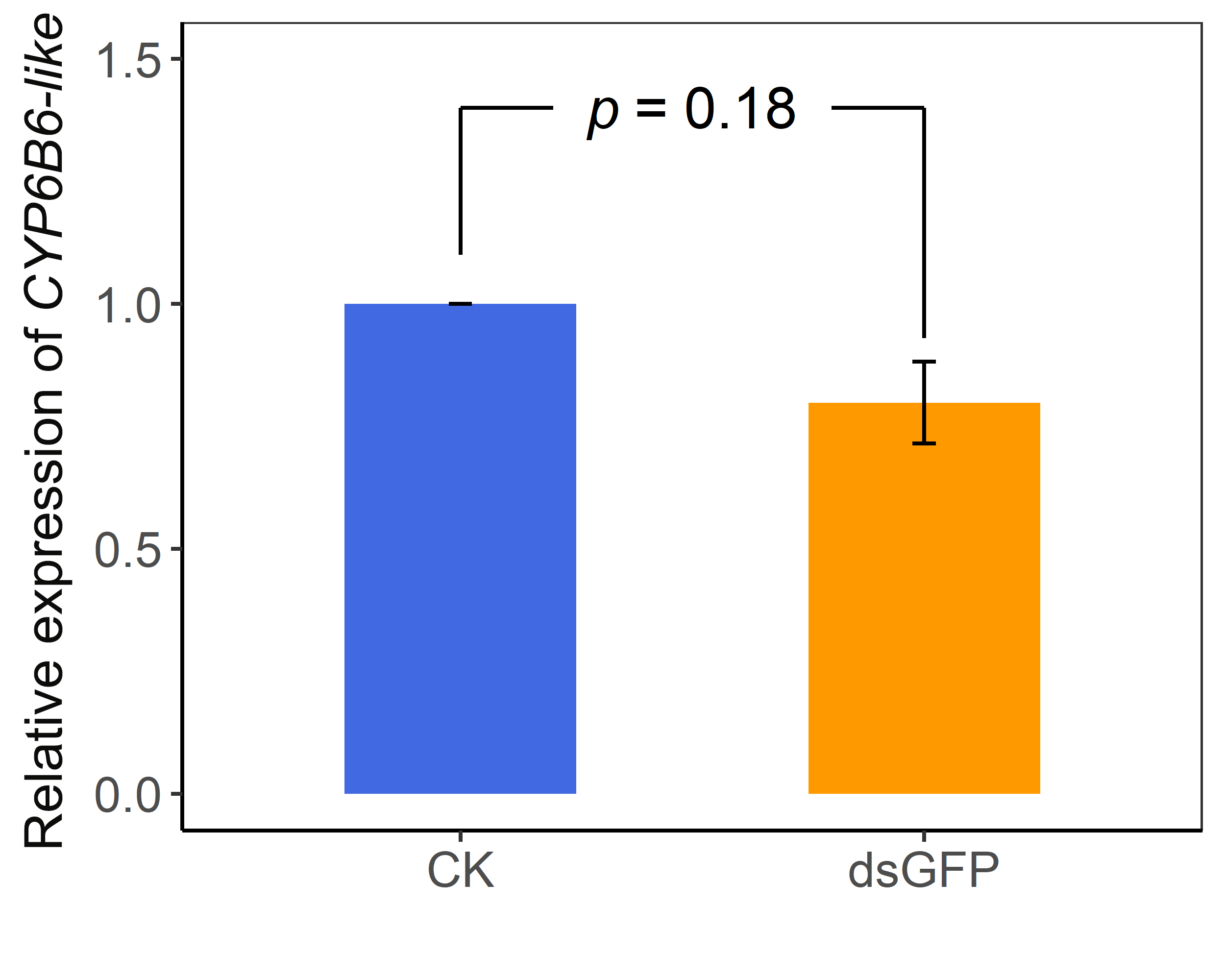

Supplement: Supplementary file 1 [file ijms-23-12245-s001.zip › Supplementary Material/S11_Fig.tif]

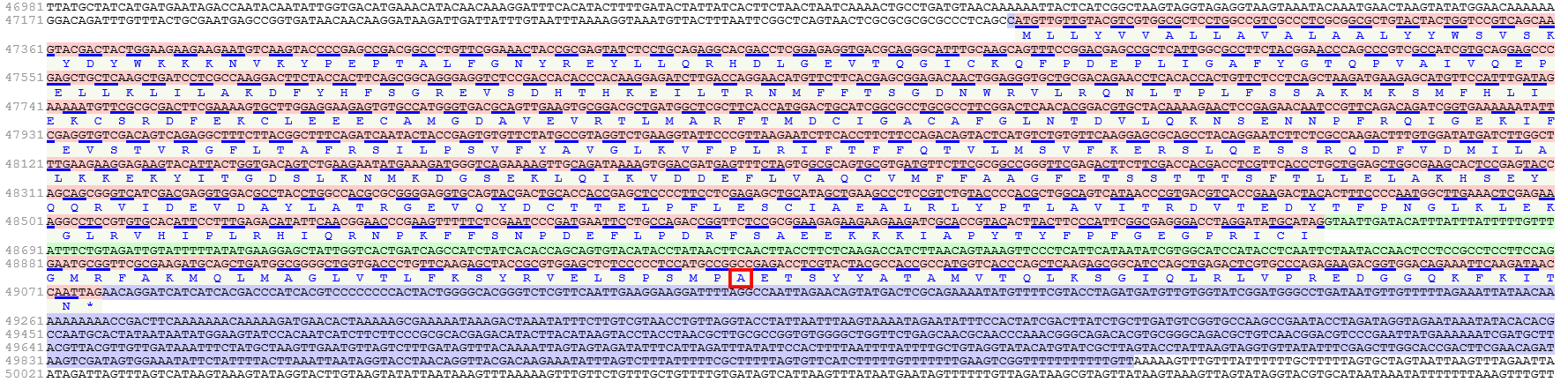

Supplement: Supplementary file 1 [file ijms-23-12245-s001.zip › Supplementary Material/S1_Fig.tif]

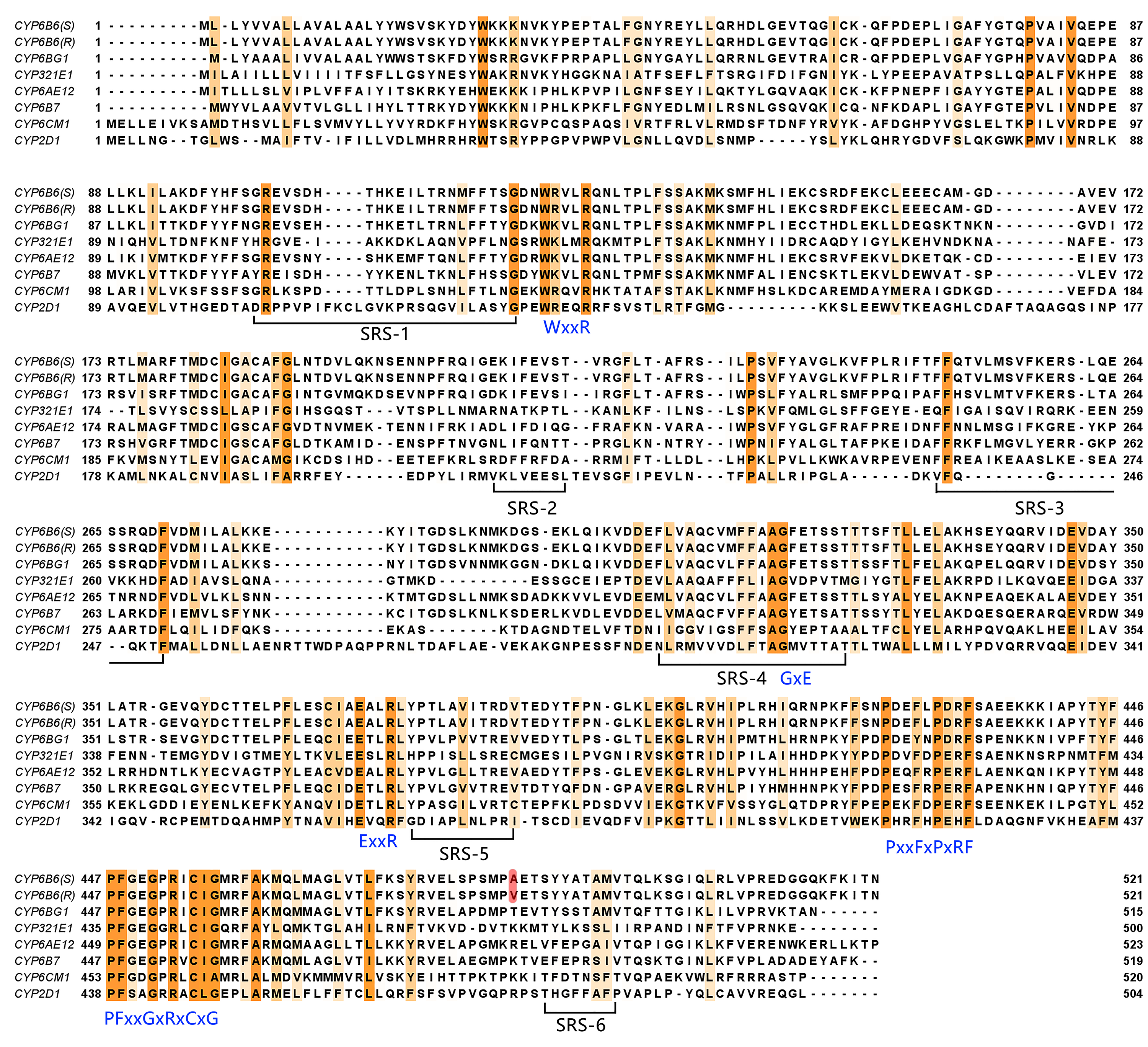

Supplement: Supplementary file 1 [file ijms-23-12245-s001.zip › Supplementary Material/S2_Fig.tif]

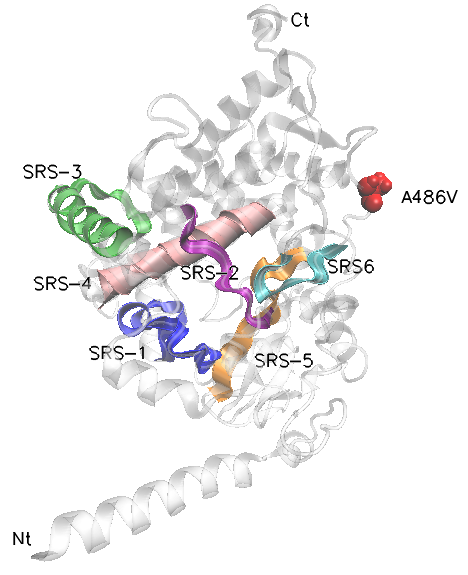

Supplement: Supplementary file 1 [file ijms-23-12245-s001.zip › Supplementary Material/S3_Fig.tif]

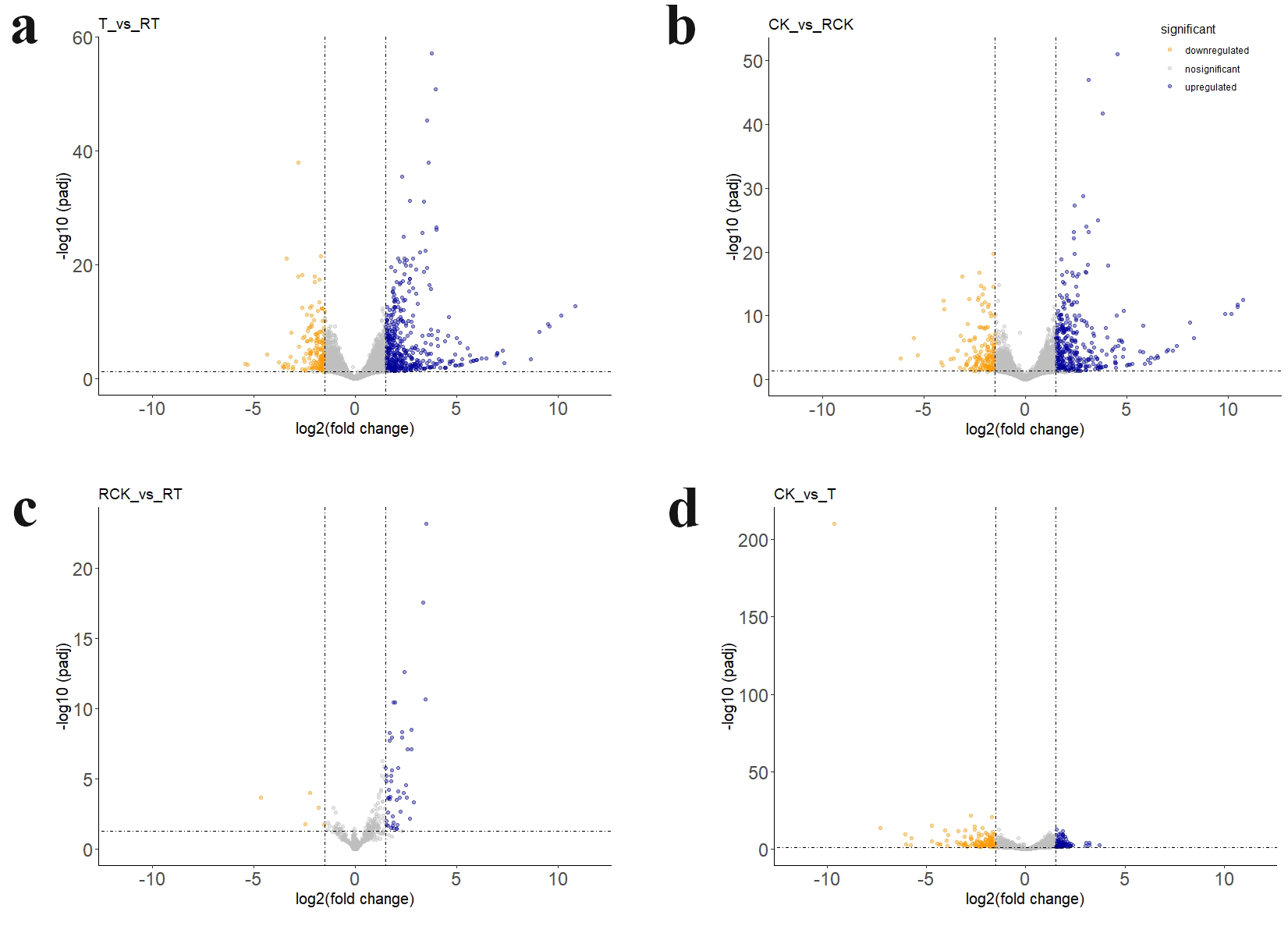

Supplement: Supplementary file 1 [file ijms-23-12245-s001.zip › Supplementary Material/S4_Fig.tif]

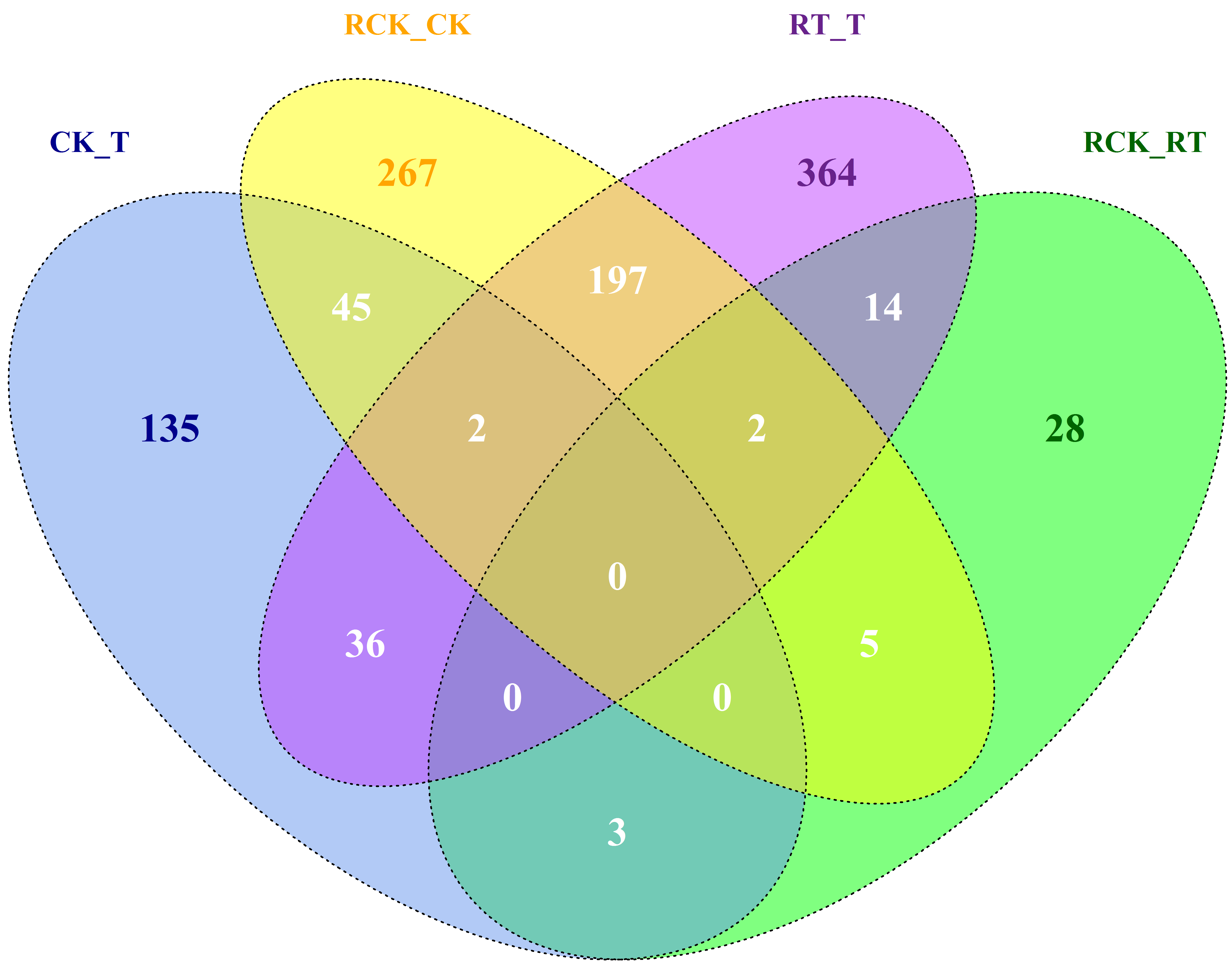

Supplement: Supplementary file 1 [file ijms-23-12245-s001.zip › Supplementary Material/S5_Fig.tiff]

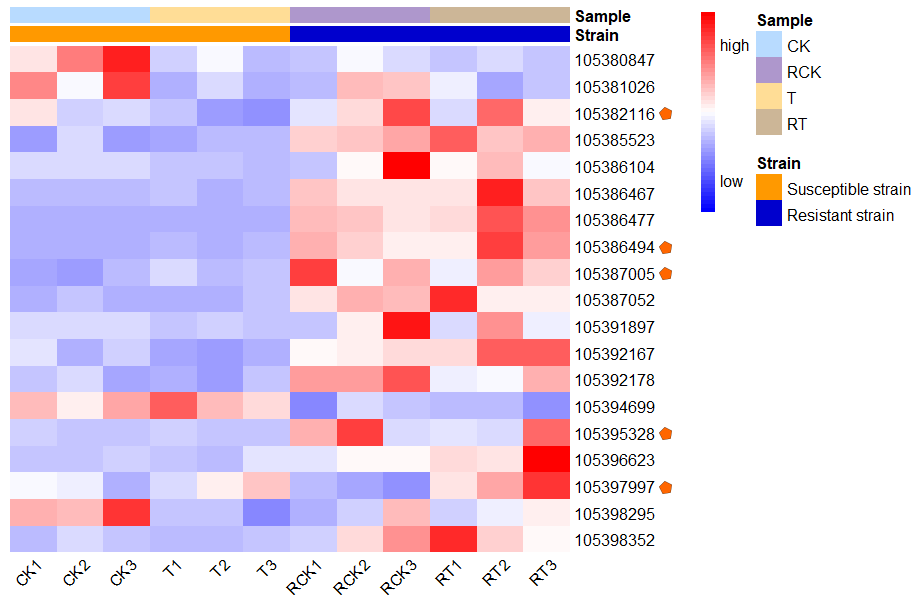

Supplement: Supplementary file 1 [file ijms-23-12245-s001.zip › Supplementary Material/S6_Fig.tiff]

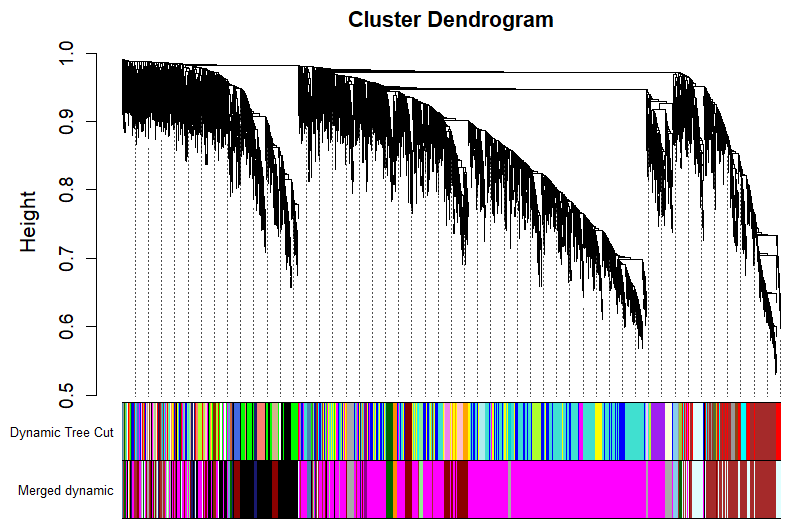

Supplement: Supplementary file 1 [file ijms-23-12245-s001.zip › Supplementary Material/S7_Fig.tif]

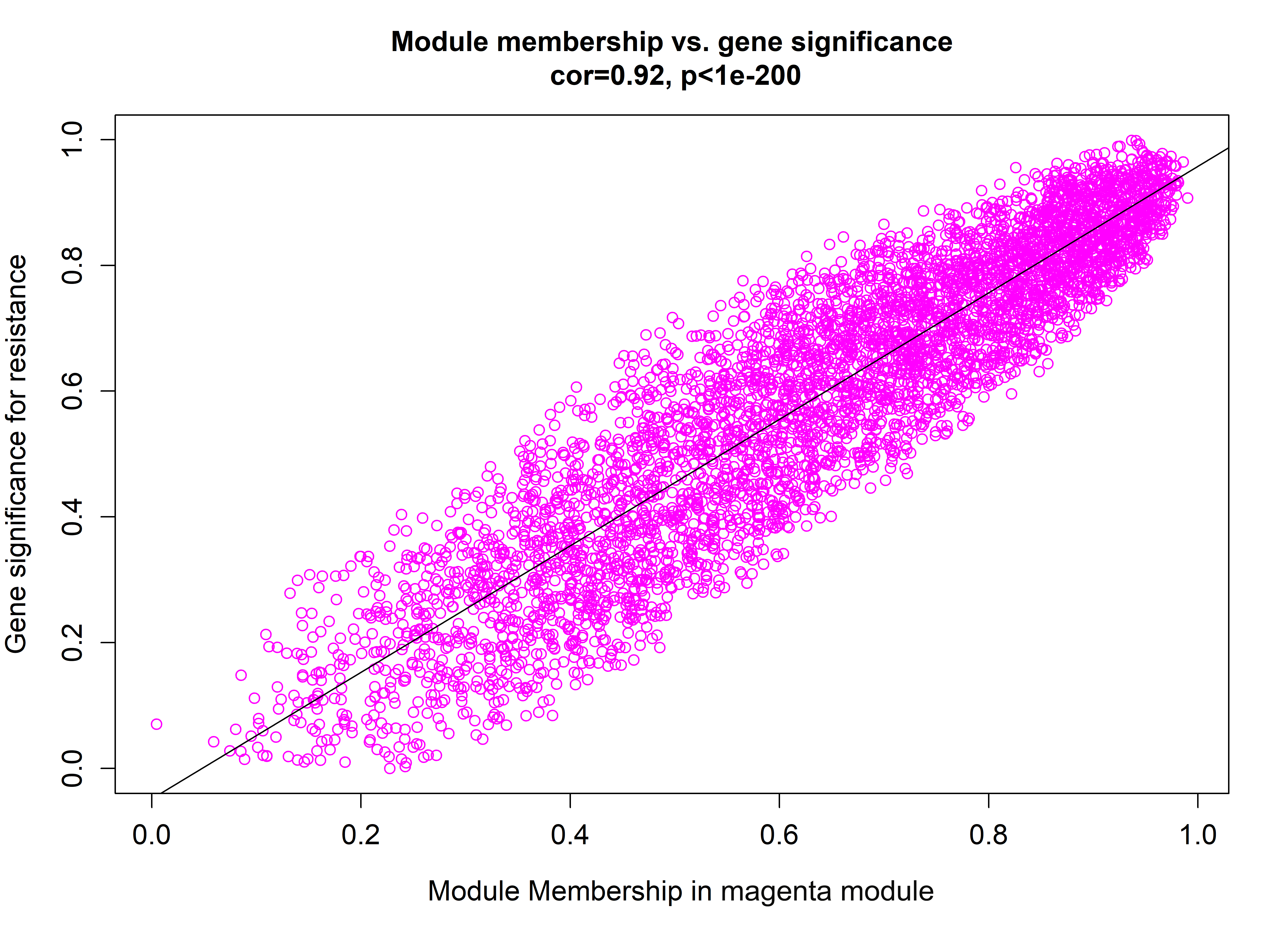

Supplement: Supplementary file 1 [file ijms-23-12245-s001.zip › Supplementary Material/S8_Fig.tif]

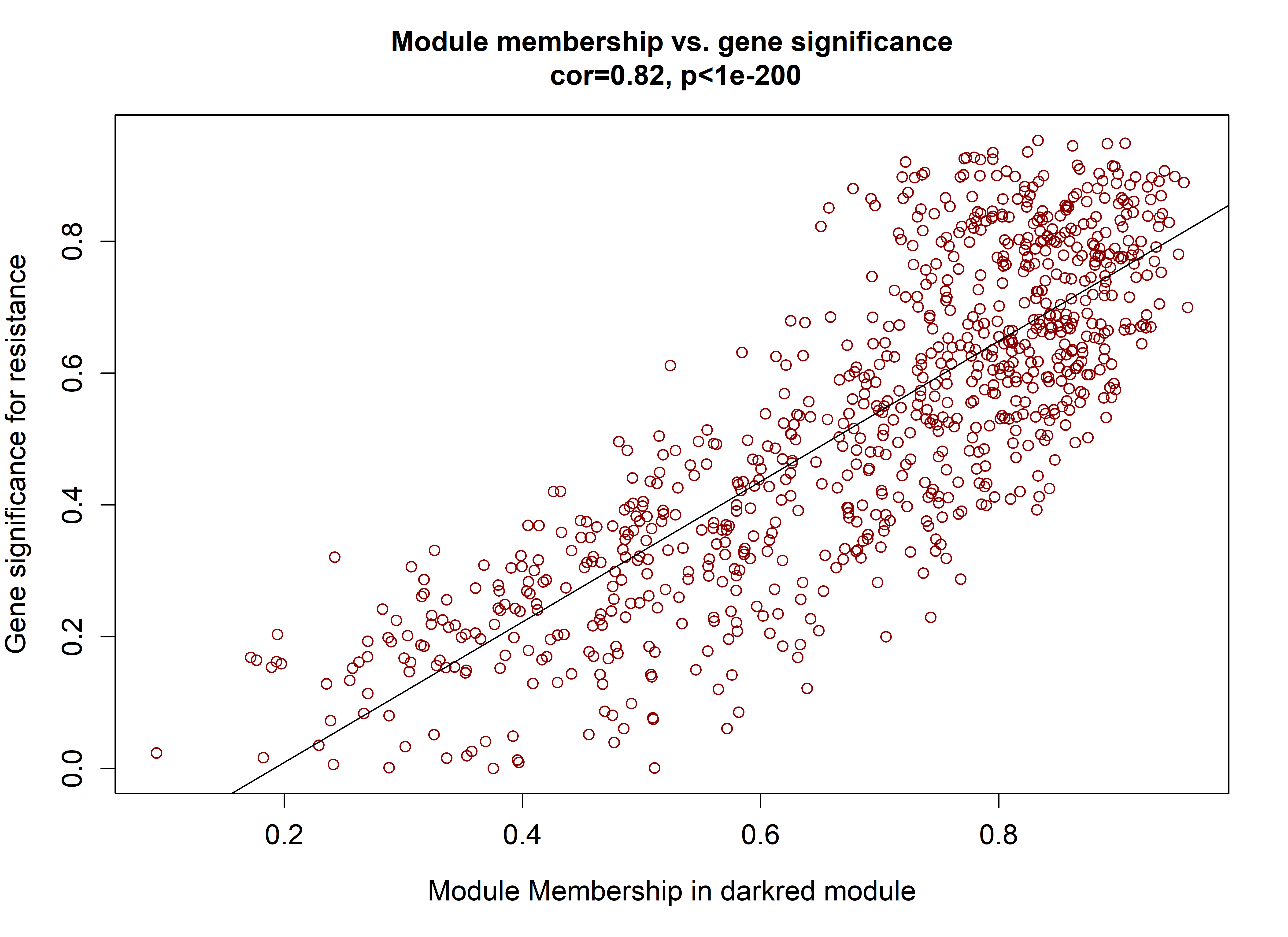

Supplement: Supplementary file 1 [file ijms-23-12245-s001.zip › Supplementary Material/S9_Fig.tif]
